# Supplementary material for: Long-Term Tailor-Made Exercise Intervention Reduces the Risk of Developing Cardiovascular Diseases and All-Cause Mortality in Patients with Diabetic Kidney Disease
Source: J Clin Med. 2023 Jan 15;12(2):691. doi: 10.3390/jcm12020691 (PMC9864356; doi:10.3390/jcm12020691)
Supplement: Supplementary file 1 [file jcm-12-00691-s001.zip › jcm-2080752-supplementary.pdf]

Figure S1 Logit conversion score and distribution of propensity scores before and after matching

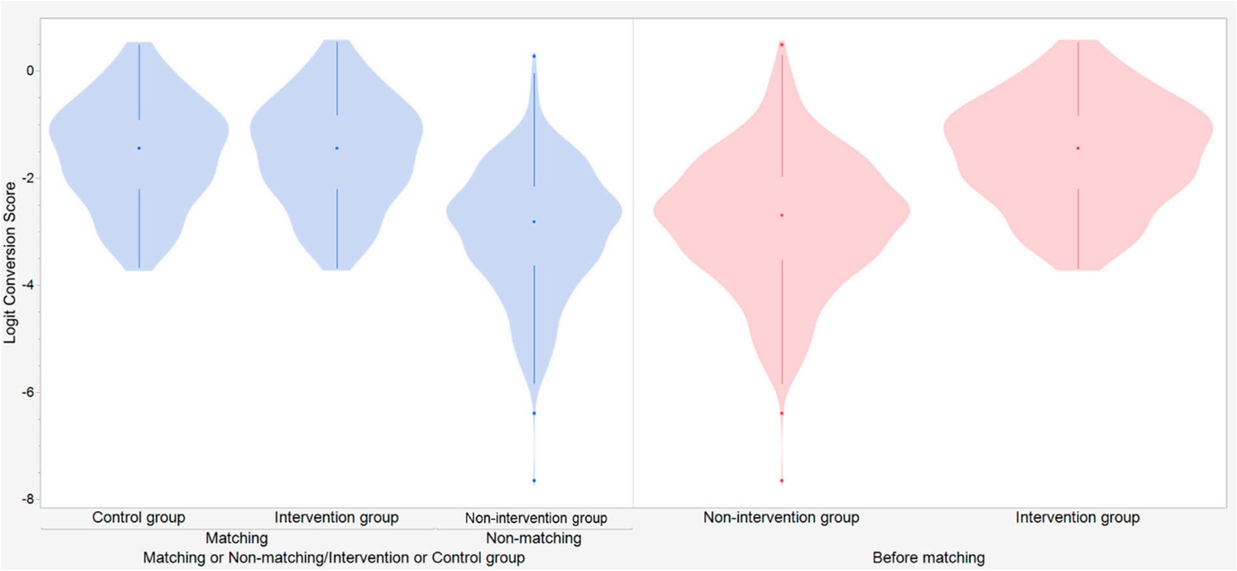

Figure S2 Changes in eGFRcr over time

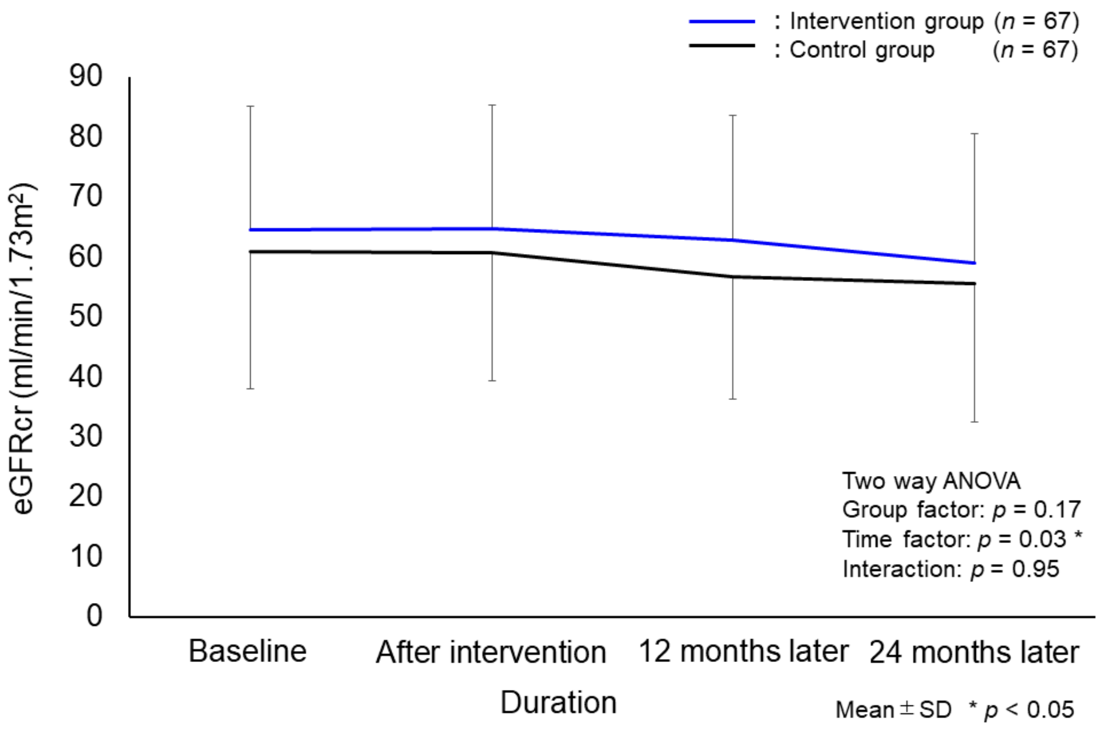

Figure S3 Changes in urinaly Alb/Cre ratio over time

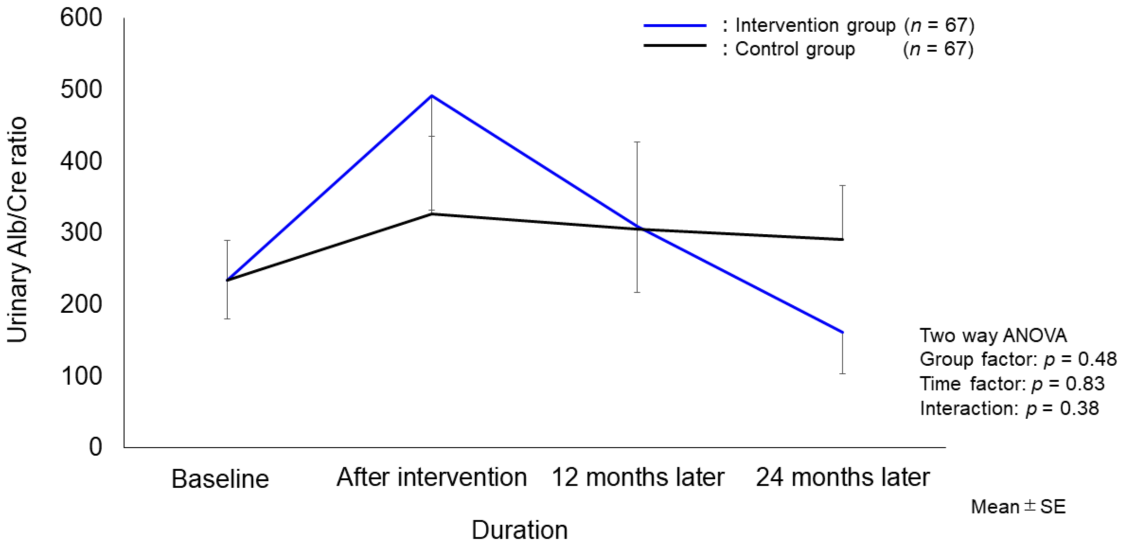

Table S1 DKD severity classification

	Albuminuria segment	A1	A2	A3
	Determination of urinary albumin<br>Urinary albumin/Creatine ratio (mg / gCr)<br>Determination of urinary protein<br>(Urinary protein / creatine ratio)<br>(g / gCr)	Normal albuminuria<br>< 30	Microalbuminuria<br>30-299	Over albuminuria<br>≧ 300<br>or<br>Highly proteinuria<br>≧ 0.5
eGFR segment (ml / min /1.73m^2)	≧ 90<br>60-89	Stage I	Stage II	Stage III
	45-59<br>30-44	Stage I	Stage II	Stage III
	15-29	Stage IV	Stage IV	Stage IV
	< 15	Stage V	Stage V	Stage V
	During dialysis therapy	Stage V	Stage V	Stage V

Table S2 Physical activity of IPAQ in the intervention group during the six months intervention preceding follow-up

	Pre intervention (n = 67)	2nd month (n = 67)	3rd month (n = 67)	4th month (n = 67)	5th month (n = 66)	6th month (n = 67)	ANOVA results
Physical activity (kcal/week)	544.9 ±1503.6	1295.1 ±1887.2	1708.8 ±2162.9 *	1750.4 ±2043.0 *	1632.9 ±1880.9 *	1344.0 ±1644.4	$p = 0.002$

Data are presented as mean ± standard deviation. One-way ANOVA and Tukey HSD were used for statistical analysis. The significance level was set at 5%. p *: vs. Pre intervention with $p < 0.05$.
